# A comparison between antenatal care quality in public and private sector in rural Hebei, China

**DOI:** 10.3325/cmj.2013.54.146

**Published:** 2013-04

**Authors:** Li Chen, Yaohua Dai, Yanfeng Zhang, Qiong Wu, Diana Rudan, Vanja Saftić, Michelle H.M.M.T. van Velthoven, Jianqiang Su, Zangwen Tan, Robert W. Scherpbier

**Affiliations:** 1 Graduate School, Peking Union Medical College, Chinese Academy of Medical Sciences, Beijing, China; 2 Department of Integrated Early Childhood Development, Capital Institute of Paediatrics, Beijing, China; 3 University Hospital Dubrava, Zagreb, and Faculty of Medicine, University of Split, Split, Croatia; 4 University Hospital “Sisters of Mercy,” Zagreb, Croatia; 5 Global eHealth Unit, Department of Primary Care and Public Health, Imperial College London, London, United Kingdom; 6 Section of Health and Nutrition, Water, Environment and Sanitation, UNICEF China, Beijing, China

## Abstract

**Aim:**

To evaluate the quality of antenatal care (ANC) in Hebei Province and compare it between the public and private sector and within the public sector.

**Methods:**

We conducted a Maternal, Newborn and Child Health Household Survey in 2010 using a two-stage sampling procedure and included 1079 mothers. The quality of ANC was assessed on the basis of the number of ANC visits, the time of the first ANC visit, 16 different ANC procedures, owning a maternal health care booklet, and the type of service provider.

**Results:**

Almost all women (98%) received ANC services at least once, 80% at least four times, and 54% at least five times. About half of the women (46%) visited ANC facility within their first trimester. Neither public nor private sector provided all 16 standardized services, but significantly more women in public sector received ANC procedures. Most women received ANC in county or higher-level hospitals (75%) and very few in township hospitals (8%). Significantly fewer women were weighed and tested for HIV/AIDS in township than in county or higher-level hospitals.

**Conclusion:**

The quality of ANC in Hebei was poorer than required by China’s national and World Health Organization norms. Although the public sector performed better than the private sector, the utilization and quality of care of ANC services in this sector varied and women generally visited county or higher-level health facilities.

To achieve universal coverage of basic public health services and increase their accessibility and affordability, the Chinese government during the health care reform in 2009 prepared a package of nine basic free public health services (Supplementary material 1(web extra material 1)) (1,2). One of the nine services was the provision of antenatal and postnatal care services (ANC, PNC). To facilitate the implementation of these services, the Ministry of Health (MoH) of China and World Health Organization (WHO) issued specific guidelines (2,3). Also in 2009, free folic acid supplementation was initiated for all rural pregnant women (4) and in 2010, MoH launched the National Action Plan for Preventing Mother-to-Child Transmission (PMTCT) of Human Immunodeficiency Virus (HIV)/Acquired Immune Deficiency Syndrome (AIDS), syphilis, and hepatitis B (HBV), aiming to integrate PMTCT into standard ANC services (5).

The private health sector in China was abolished from 1949 to the 1970s, but was revitalized in the 1980s since it was realized that it can help meet public health goals through utilizing additional health resources, increasing general access to the services, and facilitating service responsibility (6-8). From 1980 to 1990, the number of private health facilities sustained a steady growth (around 3% per year), followed by a steep rise in the early 2000s (around 40%) (8,9). However, private health facilities are often criticized for their suboptimal performance and inadequate preventive care provision (10).

Although China’s recent medical reform focuses on the private health sector, no studies have so far compared the quality of ANC care in the public and private sector. Thus, we conducted a Maternal, Newborn and Child Health (MNCH) Household Survey in two counties in Hebei Province in 2010. Our aim was to evaluate the ANC quality and compare the quality of care between public and private sectors and within public sectors in rural areas.

## Methods

### Study design and definitions

This cross-sectional survey was conducted from August 1 to 10, 2010. A standardized Maternal, Newborn and Child Health Household Survey (MNCH) survey questionnaire (11) was administered to 2406 caretakers. The coverage of ANC was defined as the percentage of women who received ANC during pregnancy among the surveyed women who had children younger than two years according to Chinese and international norms (Table 1). The quality of ANC in our study was assessed in terms of the services that pregnant women received, the number of antenatal visits, the time of the first antenatal visit, 16 different ANC procedures, owning a maternal health care booklet, and the type of ANC providers. The 16 ANC procedures are physical examinations (measuring weight and blood pressure), diagnostic tests (hemoglobin test; urine sample taken including bacteriologic examinations and tests for blood, protein, glucose, ketones, etc; syphilis, HBV test, and HIV/AIDS test) and preventive care procedures (iron supplements, folic acid supplements, advice on nutrition during pregnancy, advice on syphilis, HBV, and HIV/AIDS, advice on delaying the next pregnancy, and breastfeeding counseling). The public sector was defined as government owned or collectives-owned county or higher-level hospitals, maternal and child hospitals, township hospitals, and village clinics. The private sector was defined as non-government owned and for-profit institutions that provide health services. Based on a literature review, the characteristics of public and private sector were compared and summarized (Table 2) (12-21).

**Table 1:** Antenatal care (ANC) services received with reference to national and international policy guidelines (N = 1057)

ANC services	No. (%) of patients	National policy*	WHO’s recommendation^†^
ANC received:		Every pregnant woman should receive ANC five times during her pregnancy	Every pregnant women should receive routine ANC four times during her pregnancy
<5 times	485 (46)		
≥5 times	572 (54)		
Maternal health care booklet^‡^		Township hospitals in rural areas should distribute a maternal health care booklet to every pregnant woman in their catchment area	Not mentioned
yes	681 (64)		
no	375 (36)		
First time to receive ANC		Every pregnant woman in their catchment area should receive ANC once during her first trimester of gestation	Every pregnant woman should receive ANC once before the 4th month of pregnancy
<3 mo	491 (46)		
≥3 mo	566 (54)		
Providers^§^		All ANC providers should be certified and trained for skills needed for providing ANC	Not mentioned
doctors	903 (86)		
nurses	119 (11)		
midwifes, family planning workers, and other providers	29 (3)		
Physical examination			
weighed	861 (81)	Every pregnant woman should be weighed	Every pregnant woman should be weighed
blood pressure measured	962 (91)	Every pregnant woman should have blood pressure measured	Every pregnant woman should have blood pressure measured
Diagnostics			
hemoglobin test	830 (79)	Every pregnant woman should undergo hemoglobin test for anemia detection	Every pregnant woman should undergo hemoglobin test for anemia detection
urine sample taken	811 (77)	Every pregnant woman should undergo urine test (Urine routine test and urine protein test)	Every pregnant woman should undergo urine test (Urine routine test and urine protein test)
syphilis test	511 (48)	Every pregnant woman should undergo syphilis test	Every pregnant woman should undergo syphilis test
HBV test	625 (59)	Every pregnant woman should undergo HBV test	Every pregnant woman should undergo HBV test
HIV/AIDS test	497 (47)	Every pregnant woman should undergo HIV/AIDS test	Every pregnant woman should undergo HIV/AIDS test
Prevention			
iron supplements	235 (22)	No policy	Give all pregnant women iron once daily in pregnancy
folic acid supplements	527 (50)	Every woman in rural areas should receive free folic acid supplementation before pregnancy and during the first trimester of gestation	Give all pregnant women folic acid once daily in pregnancy
advised on nutrition during pregnancy	623 (59)	Every pregnant woman should receive counseling on nutrition during pregnancy	Every pregnant woman should receive counseling on nutrition during pregnancy
advised on birth and emergency plan	445 (42)	Every pregnant woman should receive counseling on birth and emergency plan	Every pregnant woman should receive counseling on birth and emergency plan
advised on syphilis	133 (13)	Provide counseling on changing risky sexual behavior and information on preventing transmission of syphilis, HBV and HIV/AIDS if test is negative. Further treatment and counseling if test is positive	Provide counseling on safer sex if test is negative. Further treatment and counseling if test is positive
advised on HBV	227 (21)		Not mentioned
advised on HIV/AIDS	152 (14)		Provide counseling on safer sex if test is negative. Further treatment and counseling if test is positive.
advised on delaying the next pregnancy	301 (28)	One child policy	Advice and counseling on family planning
advised on breastfeeding^II^	291 (28)	Every pregnant woman should receive counseling on breastfeeding during pregnancy	Every pregnant woman should receive counseling on breastfeeding during pregnancy

*National policy refers to Law of the People's Republic of China on population and family planning, the six major public health services, nine basic public health services, and National Action Plan for PMTCT of HIV, syphilis, and HBV (4-6,28).

†World Health Organization (WHO) recommendation refers to pregnancy, childbirth, postpartum, and newborn care: a guide for essential practice issued in 2006 (3).

‡Data on 1 patient are missing.

§Data on 6 patients are missing.

IIData on 5 patients are missing.

**Table 2:** Comparison of public and private sector in China

Similarities of public and private sector			
**Human resources**	Both can decide on employment of their physicians and payment of salaries and bonuses (12). Although multi-site practice is now allowed by the Chinese government, physicians rarely work in the public and private sector simultaneously (13).		
**Fee-for-service driven**	Both are required to raise revenue by charging patients for services. They are prone to avoid providing unprofitable (public health) services and over-prescribe the profitable high-tech diagnostic services and drugs (14).		
**Differences of public and private sector**			
	**Public sector**	**Private sector**	
**Definition**	Health sectors owned and operated by the national, provincial, city and county government, by “collectives” (eg, commune owned companies) and by the state-owned industrial enterprises (15).	Non government owned for-profit or non-profit (though very rare in China) health facilities, including individual and group practices and private hospitals (8). Private sector in China has three categories: 1) “Top-end” private providers Offer advanced medical care with high-tech equipment and charge very high prices (16). 2) Small private clinics and village doctors* Offer a mixture of traditional herbal and biomedicine services and charge low prices (16). 3) Private hospitals* Offer specialized health services for low prices, some of them were transferred from public to private hospitals (17,18).	
		**Small private clinics and village doctors**	**Private hospitals**
**Development history**	Since the establishment of new government in 1949, public sectors have played a predominant role in provision of health care (over 60%) (8,9).	Before 1949, private sector provided most of the health care services. Later, private sector was almost eradicated for 30 y. From the 1980s, the private sector revived and since mid-1990s has grown more rapidly. In 2009, the medical reform claimed to expand private sector in health service delivery in China (8).	
**Location**	Widely distributed in urban and rural areas, but more concentrated in urban areas (9).	Usually in rural towns, villages, and newly urbanized areas (16).	Distributed in urban areas and in rural counties (17).
**Health services**	Provide general acute care, and tertiary (specialized and advanced) services and remain the primary provider of outpatient traditional Chinese medicine services (14).	Serve nearly 80% of rural residents and manage diseases that are not severe and chronic diseases (19).	Often specialized, for example in stomatology, ophthalmology, gynecology, or obstetrics etc. They cater to the surgical outpatient market and have a less severe case-mix. They also play an increasing role in the ambulatory sector (14,15,17).
**Infrastructure**	More beds, asset value and outpatient and inpatient volume (14).	Fewer beds, less asset value, and outpatient and inpatient volume (14).	
**Human resources**	More staff (14).	On average one or two village doctors per clinic (19).	Fewer doctors per private hospital compared with doctors per public hospital. Elderly doctors and young nurses make up a substantial proportion of the health care workforce, because the former are retired from public hospitals and the latter are just entering the health care market (12).
**Supervision and regulation**	Within the three-tier health system, each level of public sectors receives supervision by higher level public sectors and health authorities (12,20).	Poor government regulation and poor coordination are major stumbling blocks for the development of private sector, since the responsibility for oversight was spread over many vertically structured regulatory agencies. Neither rural health administrators nor health professionals at higher-levels (eg, township and county health facilities) were providing adequate technical guidance and supervision of the village-level medical practice (15,21).	
**Service fee**	Usually higher than private village clinics or doctors and private hospitals, but lower than “top-end” private providers (14).	Low fee charged per visit (19).	Often charge prices that are lower than public hospitals and serve patients with a low education level, without insurance coverage and low or middle level income. However, private services are not included in the social insurance benefit package and patients need to pay fees themselves (14,15),
**Patient satisfaction and perception**	Low patient satisfaction. Bad staff attitudes, complicated registration procedures, and lack of responsiveness to patients’ needs. Over-prescription of drugs and diagnostic tests. However, the skills of public sectors workers are perceived to be higher and more accredited by patients (21).	High patient satisfaction. Often conveniently located and more responsive; for example, offering flexible hours and showing better attitudes toward patients (18,20,21).	

*We describe small private clinics and village doctors and private hospitals, because most of the literature focuses on these two types of private sector facilities.

### Study area

Hebei province is located in the northern part of North China Plain, with an area of 190 000 km^2^, bordering the capital city of Beijing. At the end of 2008, the total population of Hebei province was 69 890 000, with 58% of inhabitants living in rural areas. The proportion of out-migrants in search of work outside the province was 14%, which is only half of the national figure (22,23). Hebei has 11 prefectures, 172 counties, 2228 townships, and 49 216 villages. The per capita net income of rural residents in 2008 was 4796 CNY ( ≈ 760 USD); nearly the same as the national level (4761 CNY ≈ 756USD) (24,25).

The study was conducted in two counties in Hebei Province – Zhao County, located in the central part of the province, with an area of 675 km^2^, and Luannan County, situated near the Bohai Sea in the north of the province, with an area of 1270 km^2^. Both counties have comparable total populations of around 580 000, and similar per capita net income of rural residents, female illiteracy rate, and proportion of villages that have access to tap water, respectively: 5553 CNY ( ≈ 881 USD), 10.6%, and 100% in Zhao County, and 5850 CNY ( ≈ 929 USD), 11.2%, and 97% in Luannan County (20), which is better than the national level (4761 CNY, 4.08%, and 42%, respectively) (25,26).

### Sample size and sampling

The sample size was estimated using the formula n = 4*p*(1-p)*deff/e^2 (27). It was estimated that 2400 caregivers of children under five should be included in the study, which was 1200 per county. Households were selected using a two-stage sampling procedure. In the first stage, 100 villages in each county were selected using systematic sampling with a probability sampling design weighted by the total population of each village. In the second stage, the list of names of all eligible children under five years in each village was used to select 14 children. Children from each village were divided into five age groups (each representing one year interval). With preferential sampling of younger age groups, in which health problems are usually most prevalent, four children were randomly selected from 0-1 year age group, three children from 1-2 years age group, three children from 2-3 years age group, two children from 3-4 years age group, and two children from 4-5 years age group. The random numbers were generated using Excel function RAND. The list of names excluded the families that did not live in the area for at least six months. Thus, children were considered “eligible” if they were registered as permanent residents. A total of 2406 eligible households were interviewed. Only caregivers, mostly mothers, with children younger than 24 months completed the antenatal care module to reduce the recall bias.

### Tool development and training

A workshop on adaptation of the WHO generic MNCH survey tool was conducted to reach a consensus on the survey questions among experts from WHO, MoH, and maternal and child health professionals, as well as a pre-test of the survey tools in a non-project county in Hebei Province.

Study supervisors were recruited both from Capital Institute of Pediatrics and local maternal and child health hospitals. Interviewers were medical school students from the Hebei United University. Supervisors were trained for three and a half days and interviewers were trained for two days. One field practice was included in the training course. Inter- and intra-interviewer reliability for completing survey instruments after the training was assessed using a standardized video (30 minutes). The reliability was over 90% in all measurements, so we believe that all interviewers were asking or interpreting questions in the same way. Quality-control measures also required that each supervisor attended four interviews and checked every questionnaire in his or her team each survey day.

### Data management and analysis

Data were collected using a structured questionnaire (11) and data collection forms were checked for errors and completeness. Data were entered into Epidata 3.0 (The EpiData Association, Odense, Denmark) using the double entry system. Among 2406 interviewed caregivers with children under 5 years, 1079 caregivers with children under 24 months were included. Two steps of analysis were performed. The first step included all mothers with children under 24 months to assess the quality and procedure of antenatal care in general (n = 1079). The second step included only mothers who went to a single health facility to receive ANC to assess the quality and procedure of ANC in health facility settings (n = 559). χ^2^ test and Fisher exact test were used to examine the differences across health facility settings. Statistical analysis was performed using SAS 9.1 (SAS Institute, Cary, NC, USA) and α = 0.05 was considered statistically significant. The study was approved by the Ethics Committee of Capital Institute of Pediatrics, Beijing, China. All participants read the Information Consent Form and provided the written consent (Supplementary material 2(web extra material 2)).

## Results

Among 1079 surveyed mothers with children younger than 24 months, 98% reported to have received ANC at least once during their pregnancy and 80% at least four times. Fifty four percent of women received ANC at least five times, 64% owned the maternal health care booklet, 46% received ANC before 3 months of gestation, and 86% had medical doctor as a provider (Table 1). The coverage and timing of ANC deviated from the national norm that every women should receive ANC five times during her pregnancy and once during the first trimester of gestation. Eighty-one percent of women were weighed and 91% had blood pressure taken, which is close to the country’s norms of universal coverage. There were differences between our findings and national policy recommendations. The proportions of women who underwent hemoglobin, urine, syphilis, HBV, and HIV/AIDS test during pregnancy, received folic acid supplement, and were advised on nutrition during pregnancy were 79%, 77%, 48%, 59%, 47%, 50%, and 59%, respectively. A small proportion of women were given iron supplement (22%) and advice on syphilis (13%), HBV (21%), HIV/AIDS (14%), and delaying the next pregnancy (28%) (Table 1).

Among 559 mothers who received ANC in a single health facility, 448 (80%) received it in county public hospitals or higher-level facilities, 44 (8%) in township hospitals, and 64 (11%) in private sector.

More women in public than in private sector had a maternal health care booklet (74% vs 47%, *P* < 0.001) (Table 3). No differences between the public and private sector were found in maternal age, number of children younger than five years, and age and sex of children. Also there were no differences in the proportions of women who received ANC more than five times, received ANC before three months of gestation, and the type of ANC provider (Table 3).

**Table 3:** Characteristics of patients and antenatal care (ANC) in different health settings (N = 559)

	No. (%) of patients in					
Characteristics	public sector				private sector	public vs private, *P* value
	county or higher level	township level	county vs township, *P* value	total^II^		
**Maternal characteristics**						
Maternal age (years):			0.073			0.813
<25	131 (29)	20 (23)		141 (28)	21 (33)	
25-27	106 (24)	5 (11)		113 (23)	12 (19)	
28-31	132 (29)	16 (36)		149 (30)	18 (28)	
>31	79 (18)	13 (30)		92 (19)	13 (20)	
No. of children under five:*			0.003			0.750
1	216 (48)	11 (25)		227 (46)	28 (44)	
≥2	232 (52)	33 (75)		268 (54)	36 (56)	
**Children’s characteristics:**						
Age (months)			0.240			0.125
<6	80 (18)	8 (18)		88 (18)	17 (27)	
6-11	165 (37)	11 (25)		177 (36)	25 (39)	
12-23	100 (45)	25 (57)		227 (46)	22 (34)	
Sex			0.393			0.823
male	254 (57)	22 (50)		278 (56)	35 (55)	
female	194 (43)	22 (50)		217 (44)	29 (45)	
**ANC:**						
Number of ANC visits			0.014			0.378
<5 times	229 (51)	31 (70)		261 (53)	30 (47)	
≥5 times	219 (49)	13 (30)		234 (47)	34 (53)	
Maternal health care booklet:^†^			0.849			<0.001
yes	331 (74)	32 (73)		365 (74)	30 (47)	
no	116 (26)	12 (27)		129 (26)	34 (53)	
Time of the first ANC visit:			0.525			0.231
<3 mo	185 (41)	16 (36)		201 (41)	31 (48)	
≥3 mo	263 (59)	28 (64)		294 (59)	33 (52)	
Providers^:‡^			0.347			0.024
doctors	373 (83)	40 (91)		416 (84)	55 (88)	
nurses	66 (15)	3 (7)		69 (14)	4 (6)	
midwives, family planning workers, and other providers	8 (2)	1 (2)		9 (2)	4 (6)	

*Data for 3 patients are missing.

†Data for 1 patient are missing.

‡Data for 2 patients are missing.

§Fisher exact test was used to compare the difference of proportions.

IIThe numbers do not add up because one woman visited both county or higher-level hospital and township hospital.

Women who had more than one child younger than 5 years of age were more likely to go to township hospitals than primiparas (75% vs 52%, *P* = 0.003). Women who visited public facility for ANC more than five times were more likely to go to county or higher-level hospitals than township level hospitals (49% vs 30%, P = 0.014).

Significantly fewer women in private than in public sector were weighed, underwent hemoglobin, urine, syphilis, HBV, and HIV/AIDS test, were given iron supplements or advice on syphilis, HBV, and HIV/AIDS. More women who received care from county or higher-level health facilities were weighed and tested for HIV/AIDS. When combining ANC procedures together, only 7 (1%) women reported to have received all 16 services from the public sector. Not a single woman reported to have received all 16 services from township level hospitals or the private sector (Table 4).

**Table 4:** Distribution of 16 antenatal care (ANC) procedures by the type of health facility (N = 559)*

	No. (%) of patients in the					
ANC services	public sector				private sector	public vs private, *P* value
	county level or above	township level	county vs township, *P* value	total^‡^		
**Physical examination**						
1. weighed	379 (85)	30 (68)	0.004	411 (83)	45 (70)	0.011
2. blood pressure measured	410 (92)	38 (86)	0.230	451 (91)	54 (84)	0.076
**Diagnostics**						
3. hemoglobin test	368 (82)	33 (75)	0.208	403 (81)	39 (61)	<0.001
4. urine sample taken	373 (83)	32 (73)	0.063	406 (82)	38 (59)	<0.001
5. syphilis test	251 (56)	20 (45)	0.099	272 (55)	17 (27)	<0.001
6. HBV test	306 (68)	26 (59)	0.422	333 (67)	26 (41)	0.001
7. HIV/AIDS test	246 (55)	17 (39)	0.029	264 (53)	17 (27)	0.001
**Prevention**						
8.iron supplements	112 (25)	7 (16)	0.172	119 (24)	6 (9)	0.007
9. folic acid supplements	224 (50)	19 (43)	0.644	243 (49)	28 (44)	0.387
10. advised on nutrition during pregnancy	277 (62)	26 (59)	0.832	305 (62)	28 (44)	0.005
11. advised on birth and emergency plan	197 (44)	23 (52)	0.321	220 (44)	29 (45)	0.950
12. advised on syphilis	72 (16)	7 (16)	0.970	79 (16)	2 (3)	0.006
13. advised on HBV	107 (24)	11 (25)	0.835	118 (24)	7 (11)	0.018
14. advised on HIV/AIDS	82 (18)	6 (14)	0.478	88 (18)	1 (2)	0.001
15. advised on delaying the next pregnancy	120 (27)	16 (36)	0.155	137 (28)	14 (22)	0.304
16. advised on breastfeeding counseling	131 (29)	16 (36)	0.302	148 (30)	19 (31)	0.959
**Standardized services (total 16 items)**	7 (2)	0 (0)	0.517^†^	7 (1)	0 (0)	0.425^†^

*2,1,14,3,61,45,60,4,4,3,5,13,6,13,5,6 women answered “Do not know” to questions 1-16.

†Fisher exact test was used to compare the difference of proportions.

‡The numbers do not add up because one woman went to both county or higher-level hospital and township hospital.

## Discussion

In our study, 98% of women received ANC services at least once, 80% received them four times, and 54% received them five times. Only 46% of women received ANC in their first trimester. Similar results were reported in western rural China (29), but in the more developed eastern region 78.5% women received ANC at least five times and 80.5% received ANC in the first trimester (30). Regional differences have been widened during the past twenty years, which can be largely explained by economic factors (31).

To achieve universal coverage of ANC services, since 1989 MoH has implemented a series of policies and programs in China’s rural areas. A national maternal and child health routine reporting system has also been established. Over the last twenty years, with continuous policy and data support, the coverage of ANC has increased (32). Recent policies have focused on improving the health system in rural areas; specific budgets have been allocated to county, township, and village level health facilities to upgrade the equipment, develop the infrastructure, and train health workers (6). However, our study showed that neither public nor private sector provided adequate quality of ANC services according to the government and WHO standards, though public sector facilities performed better. For example, less than half of the women received advice on birth and emergency planning and less than one third received advice on breastfeeding, which may increase unwanted pregnancy and suboptimal feeding after delivery. Moreover, although pregnancy-induced hypertension syndrome (PIH), including preeclampsia and eclampsia, is the third cause of maternal deaths nationally and in rural areas (9), urine samples, important for PIH diagnosis, were taken from only 77% of women.

The proportion of women undergoing routine laboratory tests such as hemoglobin and urine tests was much higher than of those receiving other preventive services. There was also a small percentage of women receiving advice on syphilis, HBV, and HIV/AIDS. Follow-up actions (iron supplementation after hemoglobin test, counseling on PMTCT of HIV, syphilis, and HBV and advice on the next pregnancy and breastfeeding) after laboratory tests were also suboptimal.

Syphilis can cause spontaneous abortion, stillbirth, and congenital defects, but can be prevented by a comprehensive public health approach including screening, standardized treatment, counseling, and follow-up (33). During the past five years, the incidence of congenital syphilis in China has increased over 12 times (34,35). Our study found that only around half of the women received a syphilis test during pregnancy and only one in eight women was given advice on the disease. Similarly, the proportion of HIV transmission from mother to child among newly diagnosed HIV infectious cases increased from 0.1% in 1998 to 1.6% in 2007 (36,37). Although HBV transmission from mother to child did not show an increasing trend, the large number of HBV carriers (0.75 to 1.01 million) among pregnant women requires urgent measures for the prevention of vertical transmission (38).

Counseling on these infectious diseases and their treatment, together with referral of positively tested women, health education on avoiding or reducing risky behavior, safe delivery and feeding options, and follow-up are of critical importance for lowering the risk of HIV, syphilis, and HBV infection among neonates (39-45). National action plan for PMTCT of HIV, syphilis, and HBV was issued in 2010 (5). It included a standardized treatment protocol, appropriate delivery methods, and preventive care procedures for pregnant women and children. However, the evidence on integrating PMTCT into ANC is scarce and further studies are needed to show its effects on coverage, quality of care, and health outcomes (46).

Many concerns have been expressed about the quality of ANC and curative care in the private sector (7,8,10). Our study showed that the quality of ANC in the private sector was significantly worse than in the public sector in terms of physical examination, diagnostic tests, and some preventive activities.

Due to the incentive-driven preferential tax policy, it is expected that private sector in China will continue to expand (6). Experience has shown that rapid growth of private sector will bring competition for patient and curative care (47,48). However, these concerns should not prevent the government to incorporate the private sector into existing public health systems and to increase the supply of ANC. Meanwhile, regulating and improving the quality of care, especially care related to public health in the private sector, requires greater effort from the government and the use of certain mechanisms: 1) setting up the minimum standards; 2) tying competition and tender process to licensing and accreditation of private sector; 3) rewarding compliance and punishing non-compliance; 4) educating both private practitioners and service-receivers to facilitate the adoption of quality services (49-51). The Chinese government has formulated the guidelines for ANC services; the service items listed in the guidelines should serve as the minimum quality of care standards for both the public and private sector. However, a monitoring mechanism should be implemented to evaluate the compliance and a subsequent reward strategy should be developed.

In our study, only 8% women received ANC in township hospitals, 75% in county or higher-level hospitals, and 11% in the private sector. Similar results have been reported in Ningxia Province, where 63% women received the first ANC at the county level or above and 34% in township hospitals (52). According to the national norms, township level facilities should be a major provider of public health care in rural areas, while county-level maternal and child health hospitals should serve as technical support and supervisory structure for township health facilities. Township level health facilities employ doctors who received secondary school medical training (after 9 years of basic education) or junior college (after 12 years of basic education) lasting two to three years (16,49) and perform preventive care activities such as immunization of children (53). However, service utilization practices at township level do not follow the national policy directives. Patients usually choose county or higher-level over township hospitals because they believe that these hospitals provide a better quality of care, even though they are situated further away and their use requires more time and greater costs (54). Such belief is a consequence of the transformations of health care and public health systems in the early 1980s (55). Township hospitals have recently received more equipment and supplies, but they still have to receive more staff training and technical support to improve their quality of care.

A strength of our study is that it fills the literature gap on the quality of ANC in public and private sector in the rural setting in China. However, it also has some limitations. The study excluded permanent residents who had been absent from home for more than half a year, which is why our sample may not be representative of the whole study population, and generalization to other settings should be done with caution. It has been shown that the utilization of ANC services by immigrant women is not as frequent as that of permanent residents (56,57), which is why our study might have overestimated the quality of care. Also, information on ANC was based on self-reporting, which could have caused recall bias. However, we tried to minimize the recall bias by surveying only mothers of children younger than two years.

In conclusion, our study found gaps in the quality of ANC, with limited follow up of screening procedures and quality differences between levels of care, and between public and private sector clinics. It is imperative to introduce policy support and increase investments in the health sector, especially at the primary level and in rural areas. The focus of such improvements should not only be on infrastructure inputs and achieving coverage, but on continuous and systematic quality improvement accompanied by monitoring and evaluation. Apart from making such services affordable and accessible, minimum standards of quality of ANC should be set up for the whole health sector, public and private alike, together with training and technical support. Finally, patients should be educated on access to ANC services and the type of services that they can expect from health facilities.
