# Supplementary material for: A comparison between antenatal care quality in public and private sector in rural Hebei, China
Source: Croat Med J. 2013 Apr;54(2):146–56. doi: 10.3325/cmj.2013.54.146 (PMC3641873; doi:10.3325/cmj.2013.54.146)
Supplement: Supplementary material 1 [file CroatMedJ_54_s001.pdf]

Supplementary material 1

Nine public health services are divided into three categories based on characteristics of population and diseases:

Public health services targeting general population

- Establish uniform and standardized health record for all resident population.
- Provide health education, health communication information and counseling services to all resident population.

Public health services targeting priori population

- Establish a health care booklet for all infant and young children aged 0-36 months and conduct a neonatal visit and systematic management for child health care.
- Provide at least five antenatal care visits for all pregnant women and two postnatal care visits for all new mothers.
- Provide health guidance to elderly population aged 65 years or above.

Public health services to prevent and control diseases

- According to National Immunization Programs, immunize all children with hepatitis B, BCG and polio vaccination.
- Timely detect, register and report patients with or suspected with infectious diseases, participate in management of epidemic-stricken places, implement health communication and counseling services on treatment and prevention of infectious diseases
- Provide guidance to high risk population for chronic diseases such as hypertension and diabetes. Register and regularly follow-up patient with diagnosis of hypertension and diabetes.
- Register and manage patients with severe mental illness. Treat, follow-up and rehabilitate severe mental illness patients living at home under the guidance of professional health organizations.

Six major public health service programs and their aims:

1. Giving booster shots of the Hepatitis B vaccine to those under the age of 15.
2. A screening program for breast and cervical cancers for women in rural areas.
3. Supplement folic acid as to prevent neural tube defects. Folic acid will be offered for free to rural women before pregnancy and in early pregnancy nationwide.
4. Helping millions of impoverished Chinese patients cure cataracts."
5. Eliminating coal-burning endemic fluorosis in six provinces including Guizhou and Yunnan. It will speed up the process that enables all the coal-burning stoves in endemic fluorosis hit regions will be replaced.
6. Renovating water supply and lavatories in rural areas. It plans to complete the renovation for 4.11 million households in 2009. At the same time, safety of drinking water will be ensured.
