# Supplementary material for: A comparison between antenatal care quality in public and private sector in rural Hebei, China
Source: Croat Med J. 2013 Apr;54(2):146–56. doi: 10.3325/cmj.2013.54.146 (PMC3641873; doi:10.3325/cmj.2013.54.146)
Supplement: Supplementary material 2 [file CroatMedJ_54_s002.pdf]

CONSENT FORM

Hello. My name is ____________. I am a medical student from Hebei United University. We are conducting a survey supported by World Health Organization. I would like to ask you some questions about you and your child. The information we collect will help the government to understand the status quo of health service and to promote health services that could improve the health of children. The questions usually take about 30 minutes. All of the answers you give will be confidential and will not be shared with anyone other than members of our survey team.

You don't have to be in the survey, but we hope you will agree to answer the questions since your views are important. If I ask you any question you don't want to answer, just let me know and I will go on to the next question or you can stop the interview at any time.

Now do you have any questions?

SIGNATURE OF INTERVIEWER:_____________DATE: _________

1. Agree to participate 2. Do not agree to participate

SIGNATURE OF INTERVIEWEE:_____________DATE: _________
